# Uniportal Video-Assisted Thoracoscopic Surgery (VATS) Lingular Segmentectomy for Solitary Pulmonary Metastasis From Endometrial Carcinoma: A Case Report

**DOI:** 10.7759/cureus.89371

**Published:** 2025-08-04

**Authors:** Oscar Carrillo, Daniela Saenz, Edwin Barrientos

**Affiliations:** 1 Department of Research, Universidad de San Carlos de Guatemala, Guatemala City, GTM; 2 Department of Medical Research, Universidad Francisco Marroquín, Guatemala City, GTM; 3 Department of Thoracic Surgery, Instituto Guatemalteco de Seguridad Social, Guatemala City, GTM

**Keywords:** endometrial adenocarcinoma, pulmonary metastasis, segmentectomy, thoracic surgery, vats

## Abstract

Endometrial adenocarcinoma frequently metastasizes to distant organs, with the lungs being a common site. Pulmonary metastases typically present as multiple nodules. However, solitary lesions are uncommon and may offer surgical opportunities. This case report describes a 42-year-old woman with a history of stage IV endometrial adenocarcinoma who was found to have a solitary hypermetabolic pulmonary nodule on surveillance by Positron Emission Tomography/Computed Tomography (PET-CT). She underwent a uniportal video-assisted thoracoscopic surgery (VATS) anatomical segmentectomy of the left lingular segments (S4-S5). Histopathology confirmed moderately differentiated metastatic adenocarcinoma of endometrial origin with clear surgical margins. This case highlights the feasibility and oncologic effectiveness of uniportal VATS segmentectomy in managing isolated pulmonary metastases from gynecologic malignancies, emphasizing the role of minimally invasive surgery in selected patients.

## Introduction

Endometrial carcinoma is among the most prevalent malignancies of the female reproductive tract, particularly in developed countries, with a steadily increasing global incidence [1]. While most cases are diagnosed at an early stage and exhibit favorable outcomes, advanced disease poses significant clinical challenges due to its potential for recurrence and distant spread. The typical patterns of dissemination include local invasion and lymphatic spread. However, hematogenous metastasis, particularly to the lungs, is less common and often associated with poorer prognosis [2,3]. Pulmonary metastases from endometrial cancer are usually multiple and bilateral, making a solitary pulmonary nodule a rare but clinically important finding [2]. Solitary pulmonary metastases from endometrial cancer have been reported in less than 5% of cases with pulmonary involvement, underscoring their rarity and clinical significance [4].

Recent advancements in thoracic surgery, particularly the adoption of minimally invasive techniques such as video-assisted thoracoscopic surgery (VATS), have expanded the therapeutic options for select patients with isolated pulmonary metastases. VATS enables direct visualization of the pleural cavity and lung parenchyma through small incisions, facilitating precise lesion localization and resection with reduced surgical trauma. In the setting of isolated pulmonary metastasis, VATS allows for both diagnostic tissue sampling and curative-intent resection, while minimizing postoperative complications and preserving lung function. Anatomical segmentectomy offers a parenchyma-sparing alternative to lobectomy, which is especially valuable in patients with limited pulmonary reserve or lesions in surgically favorable locations. Uniportal VATS, the latest evolution in minimally invasive thoracic surgery, provides comparable oncologic outcomes while reducing postoperative pain, hospital stay, and recovery time, marking a significant shift in the management of pulmonary metastases across cancer types.

## Case presentation

A 42-year-old female patient was referred to the thoracic surgery outpatient clinic following an incidental finding on routine surveillance imaging. She had a history of stage IV endometrial adenocarcinoma diagnosed in 2019, treated with total hysterectomy, nine cycles of chemotherapy, 23 fractions of external beam radiotherapy, and three sessions of high-dose-rate brachytherapy. A complete response was documented by the end of 2019.

**Figure 1:**
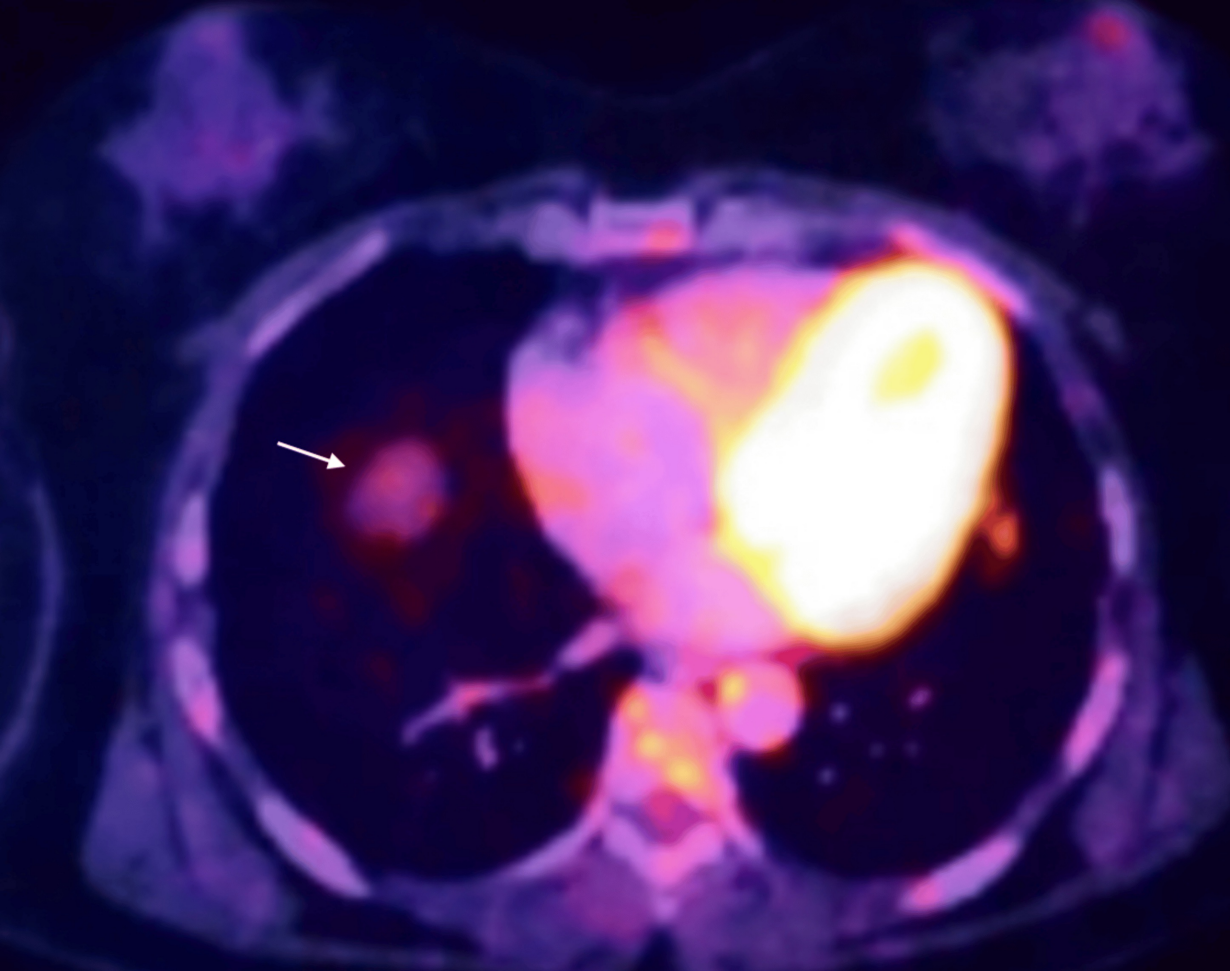
PET scan showing a suspicious hypermetabolic nodule (white arrow) in the left lung field PET: Positron Emission Tomography

Since remission, the patient had undergone annual follow-up with Positron Emission Tomography Computed Tomography (PET-CT) and laboratory testing. In her 2022 surveillance PET-CT, a hypermetabolic pulmonary nodule was identified in the left upper lobe, corresponding to the lingular segment, measuring 3 cm in diameter with well-defined borders and no associated lymphadenopathy (Figure 1).

A dedicated systemic workup and mediastinal staging with PET-CT and contrast-enhanced CT ruled out extrathoracic disease or nodal involvement, supporting the feasibility of curative-intent resection. She remained asymptomatic and was referred to thoracic surgery to evaluate surgical options. 

**Figure 2:**
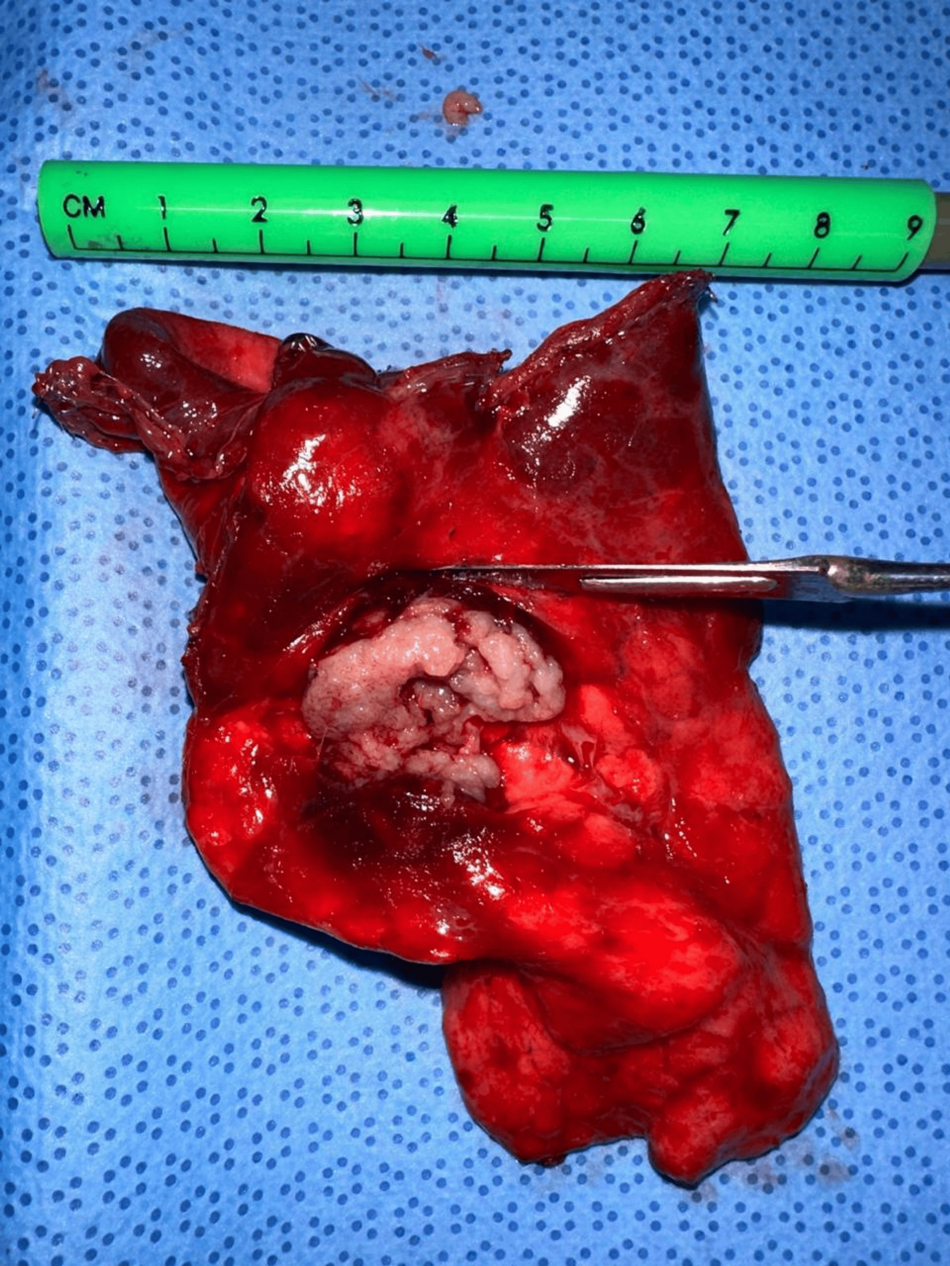
Lung segment (9 x 6.5 x 3 cm), showing a firm, well-circumscribed subpleural nodular lesion (2.8 x 5 x 5.1 cm)

After thorough evaluation and multidisciplinary discussion, the patient underwent a uniportal VATS anatomical segmentectomy of the left lingular segments (S4-S5). Under general anesthesia with single-lung ventilation, a single incision was made in the left fifth intercostal space. A well-circumscribed 3 cm lesion was identified in the lingula, adjacent to segmental pulmonary vessels. Due to its central location, anatomical segmentectomy was favored to ensure clear margins (Figure 2).

The procedure was completed without complications or conversion to thoracotomy. A thoracostomy tube was placed, and the incision was closed.

**Figure 3:**
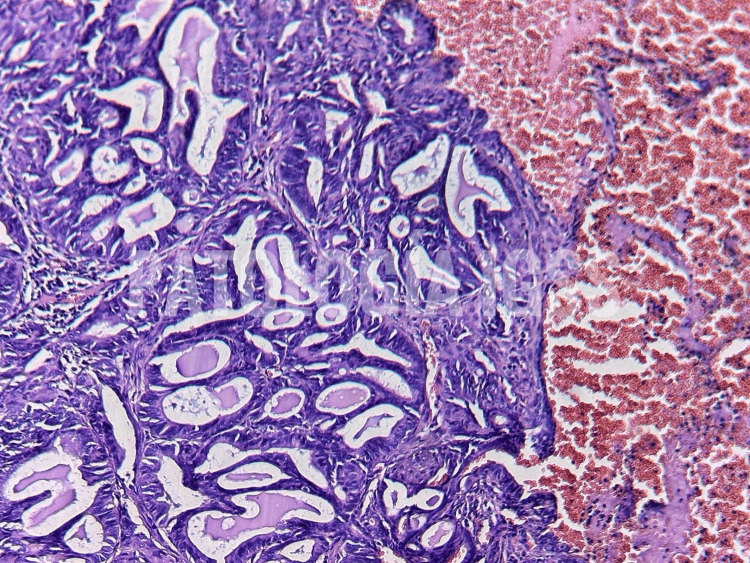
Histopathological section under Hematoxylin and eosin (H&E) staining Shows a moderately differentiated adenocarcinoma characterized by glandular crowding, nuclear pleomorphism, and a desmoplastic stromal reaction.

**Figure 4:**
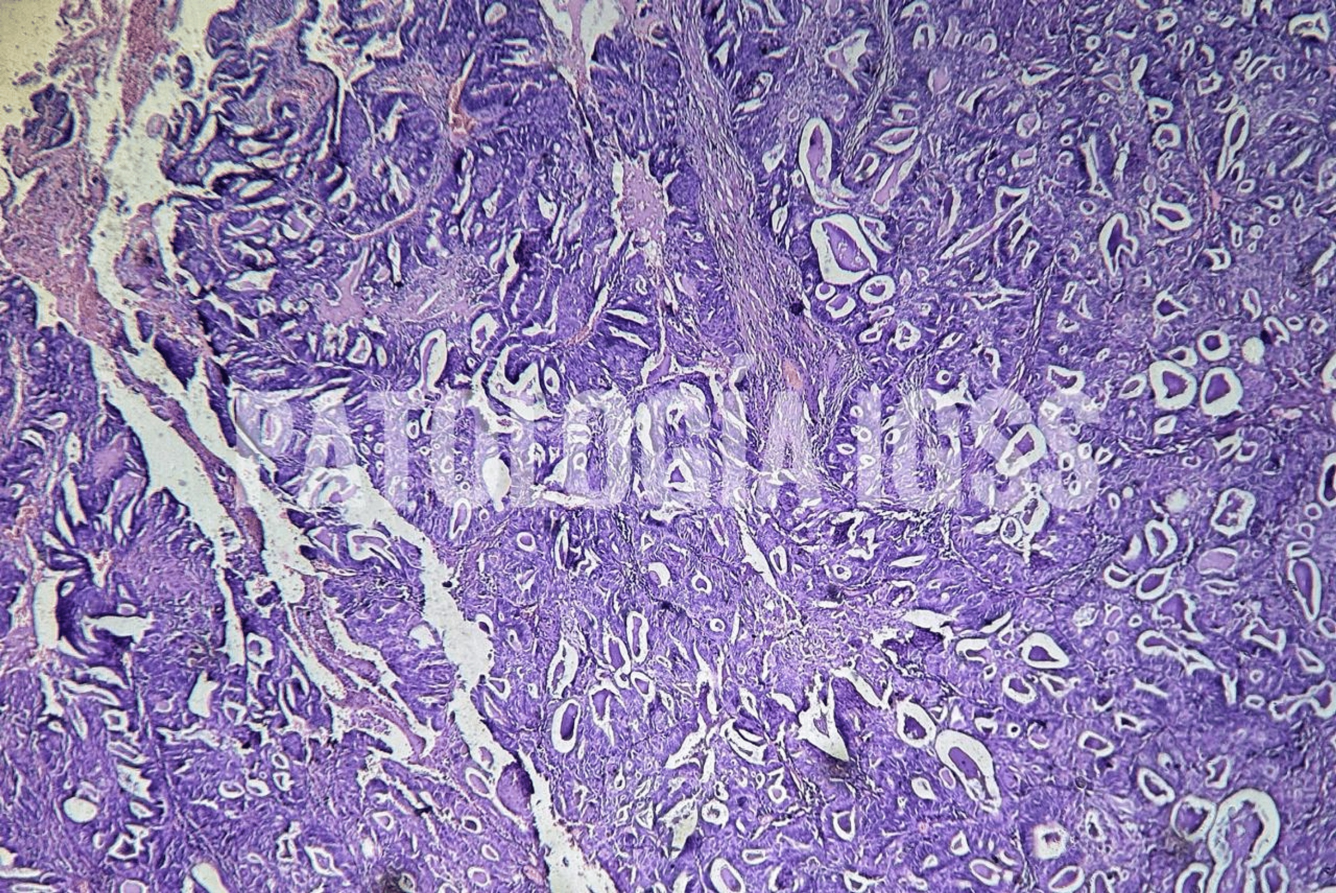
Moderately differentiated adenocarcinoma under Hematoxylin and eosin (H&E) staining Demonstrates extensive infiltration of the tissue by irregular, variably sized neoplastic glands dispersed throughout the tumor mass.

Postoperatively, the patient was extubated in the OR and monitored in the ICU. Recovery was uneventful, the chest tube was removed on day three, and she was discharged on day five without oxygen support. Histopathology confirmed moderately differentiated metastatic adenocarcinoma (Figures 3, 4) of endometrial origin, with negative margins and no lymphovascular invasion.

Pathological staging corresponded to recurrent International Federation of Gynecology and Obstetrics (FIGO) stage IVB endometrial carcinoma with solitary pulmonary metastasis (rM1a), resected with curative intent. The patient remains in regular follow-up with no signs of recurrence.

## Discussion

Endometrial carcinoma is the most common gynecologic malignancy worldwide and a significant cause of cancer-related morbidity and mortality in women [1]. While the majority of cases are diagnosed at an early stage with favorable prognosis, advanced-stage disease, such as stage IV as in this patient, carries a higher risk of recurrence and metastasis despite aggressive multimodal treatment [2,3]. Pulmonary metastases from endometrial carcinoma, although not the most frequent site, represent a critical clinical challenge due to their impact on survival and quality of life [4,5]. Typically, these metastases manifest as multiple nodules, making surgical intervention less feasible; however, isolated pulmonary metastases, as in this case, offer an opportunity for a potentially curative resection [6].

The biological behavior of endometrial carcinoma varies by histologic subtype and molecular profile, influencing metastatic patterns and therapeutic responses [3]. The predominance of the hematogenous spread to the lungs underscores the importance of vigilant surveillance post-treatment, especially in high-risk patients [5]. Prolonged disease-free intervals are associated with improved post-metastasectomy outcomes [7].

Surgical management of pulmonary metastases has evolved significantly over the past decades. Metastasectomy, though historically considered primarily palliative, now offers curative potential in selected patients, with survival benefits documented across multiple tumor types [7,8]. The criteria for surgical candidacy include controlled primary tumor, absence of extrathoracic disease, resectability of all lung lesions, and adequate cardiopulmonary reserve [2,8]. In gynecologic cancers, including endometrial carcinoma, pulmonary metastasectomy has shown promising outcomes in selected patients, with retrospective series demonstrating long-term survival in those with limited disease burden and prolonged disease-free intervals [12,13]. Such findings support the growing role of surgery beyond palliative intent in this population. This case fulfills these criteria, making the patient an ideal candidate for surgical resection.

In this context, metastasectomy provided both a diagnostic and therapeutic advantage, confirming histologic recurrence and achieving negative surgical margins. The patient’s excellent postoperative recovery and absence of recurrence to date further support the value of this approach in select individuals. Moreover, this case adds to the limited but expanding literature supporting the role of metastasectomy in patients with endometrial cancer with isolated pulmonary recurrence, particularly when performed with curative intent using minimally invasive techniques.

The uniportal VATS approach represents an important advancement in thoracic surgery, combining minimally invasive techniques with effective oncologic resection [9]. Compared to traditional open thoracotomy or multiport VATS, uniportal VATS offers reduced postoperative pain, shorter hospital stays, faster recovery, and improved cosmetic outcomes without compromising surgical efficacy [9,10]. This approach requires high technical skill due to limited access and visualization but offers precise anatomical dissection, especially important in segmentectomies where preservation of healthy lung tissue is crucial.

Anatomical segmentectomy, particularly of the lingular segments (S4 and S5), is a technically demanding procedure given the complex vascular and bronchial anatomy in this area [11]. Performing it via uniportal VATS further elevates the technical challenge but offers the benefit of preserving lung parenchyma compared to lobectomy, which is significant in patients who may require further pulmonary interventions or who have limited pulmonary reserve [10,11]. In this case, the lesion's central location near segmental vessels necessitated anatomical resection to ensure negative margins and reduce recurrence risk, underscoring the oncologic rigor achievable with minimally invasive techniques.

## Conclusions

This case underscores the growing role of thoracic surgery, particularly uniportal VATS anatomical segmentectomy, as a safe, effective, and minimally invasive option for managing isolated pulmonary metastases of gynecologic origin. It highlights how careful patient selection, long-term surveillance, and multidisciplinary collaboration can lead to curative outcomes even in advanced-stage disease. By illustrating a valuable example of technical feasibility and oncologic adequacy, this report adds to the limited literature on minimally invasive resection of endometrial cancer metastases and supports the expansion of the surgical paradigm in metastatic gynecologic oncology.
